# Supplementary material for: Aging impairs cold-induced beige adipogenesis and adipocyte metabolic reprogramming
Source: bioRxiv. 2024 Feb 1:2023.03.20.533514. Originally published 2023 Mar 23. Preprint. [Version 2] doi: 10.1101/2023.03.20.533514 (PMC10055201; doi:10.1101/2023.03.20.533514)
Supplement: Supplement 1 [file NIHPP2023.03.20.533514v2-supplement-1.pdf]

bioRxiv preprint doi: https://doi.org/10.1101/2023.03.20.533514; this version posted February 1, 2024. The copyright holder for this preprint (which was not certified by peer review) is the author/funder, who has granted bioRxiv a license to display the preprint in perpetuity. It is made available under aCC-BY 4.0 International license.

431 **Supplemental Figures**

Figure S1

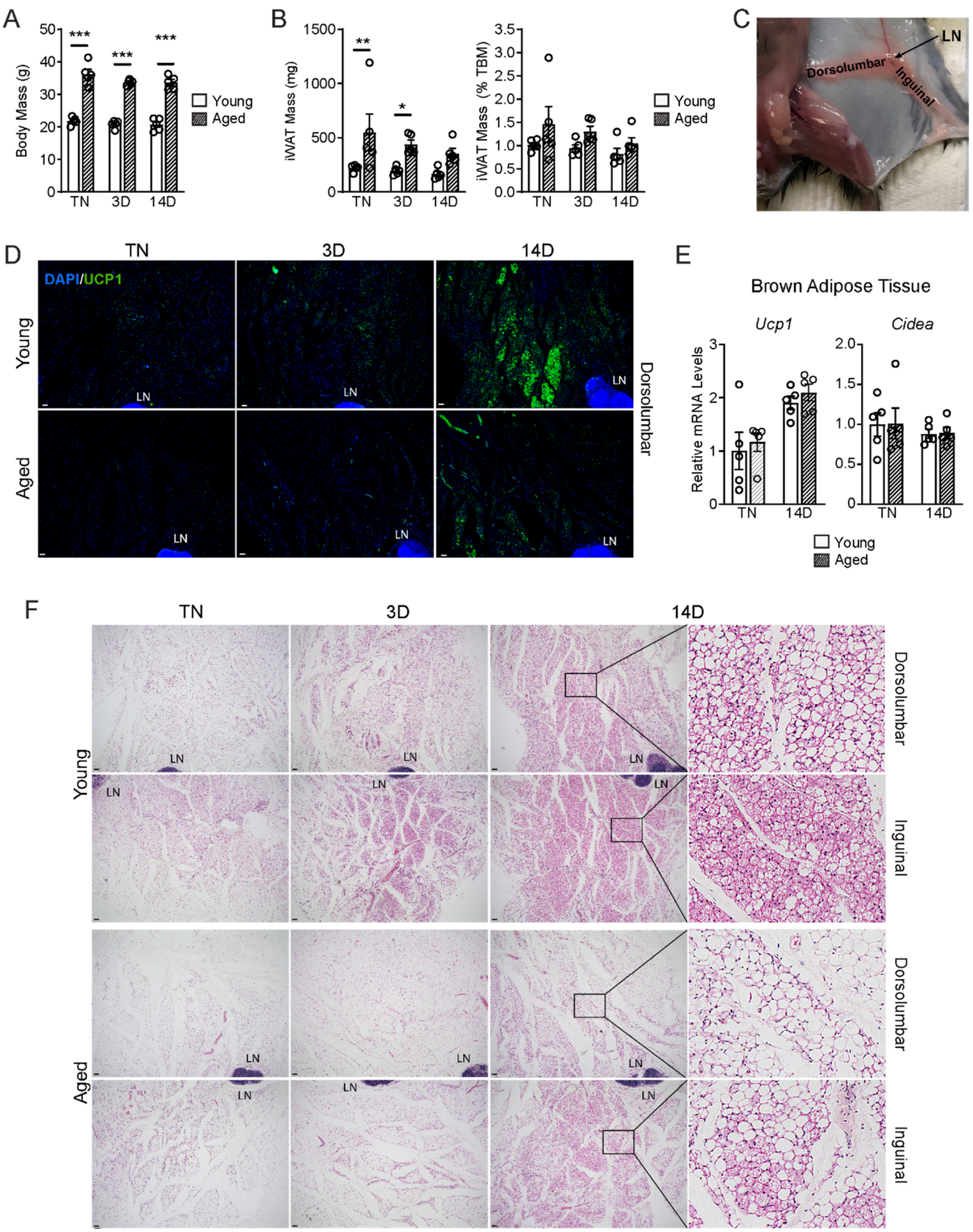


432

bioRxiv preprint doi: https://doi.org/10.1101/2023.03.20.533514; this version posted February 1, 2024. The copyright holder for this preprint (which was not certified by peer review) is the author/funder, who has granted bioRxiv a license to display the preprint in perpetuity. It is made available under aCC-BY 4.0 International license.

**Figure S1, related to Figure 1.** (**A-B**) Body mass and iWAT mass of mice described in Figure 1A, n=5. (**C**) Mouse dissection with lymph node (LN) orientation showing the dorsolumbar and inguinal regions of the iWAT pad. (**D**) Immunofluorescence analysis of iWAT with UCP1 (green) and DAPI (blue). LN=lymph node. Scale bar 100 μm. (**E**) mRNA levels of *Ucp1* and *Cidea* in BAT of young and aged mice housed at TN, and either maintained at TN or exposed to cold for 2 weeks. (**F**) H&E staining of serial sections of iWAT from D (above) and Figure 1C, LN=lymph node. Scale bar 100 μm. Data represent mean ± SEM, points represent biological replicates, analyzed using a Student's t-test with a two-way ANOVA with a Tukey correction for multiple comparisons. Significance: not significant, P > 0.05; * P < 0.05 ** P < 0.01; *** P < 0.001.

Figure S2

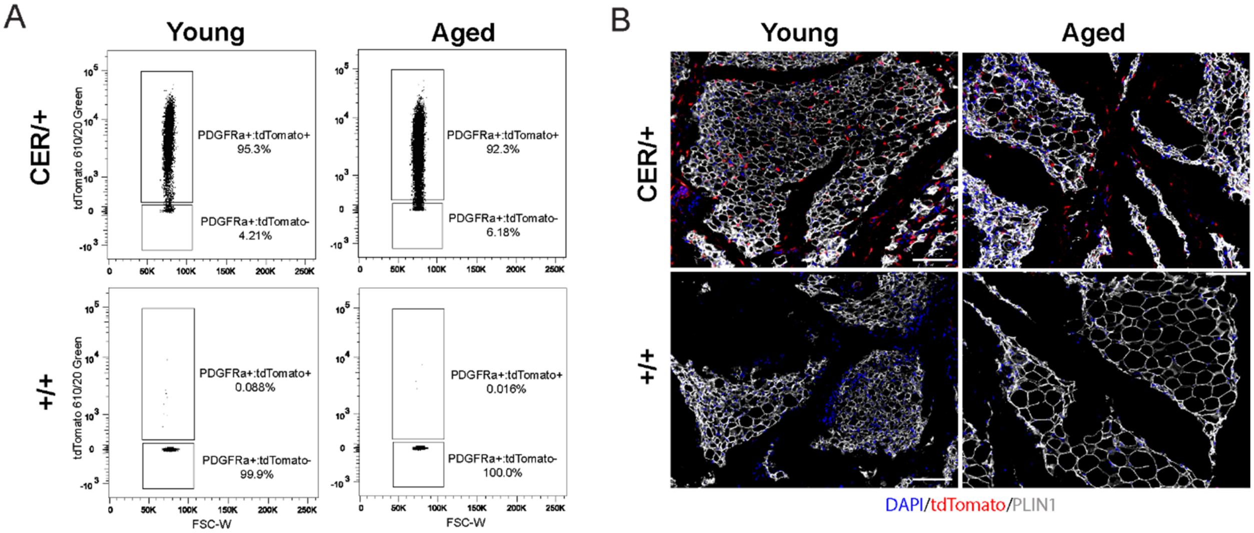


**Figure S2, related to Figure 2.** (**A**) Representative flow cytometry plots showing expression of tdTomato in gated Live, $Lin^{-}$; $PDGFR\alpha^{+}$ stromal vascular cells isolated from young and aged reporter mice (described in Figure 2) immediately after treatment with tamoxifen (tmx, pulse). (**B**) Immunofluorescence analysis of iWAT from young and aged reporter mice with tdTomato (red), PLIN1 (white) and DAPI (blue) after the tmx pulse, scale bar 100 μm.

bioRxiv preprint doi: https://doi.org/10.1101/2023.03.20.533514; this version posted February 1, 2024. The copyright holder for this preprint (which was not certified by peer review) is the author/funder, who has granted bioRxiv a license to display the preprint in perpetuity. It is made available under aCC-BY 4.0 International license.

Figure S3

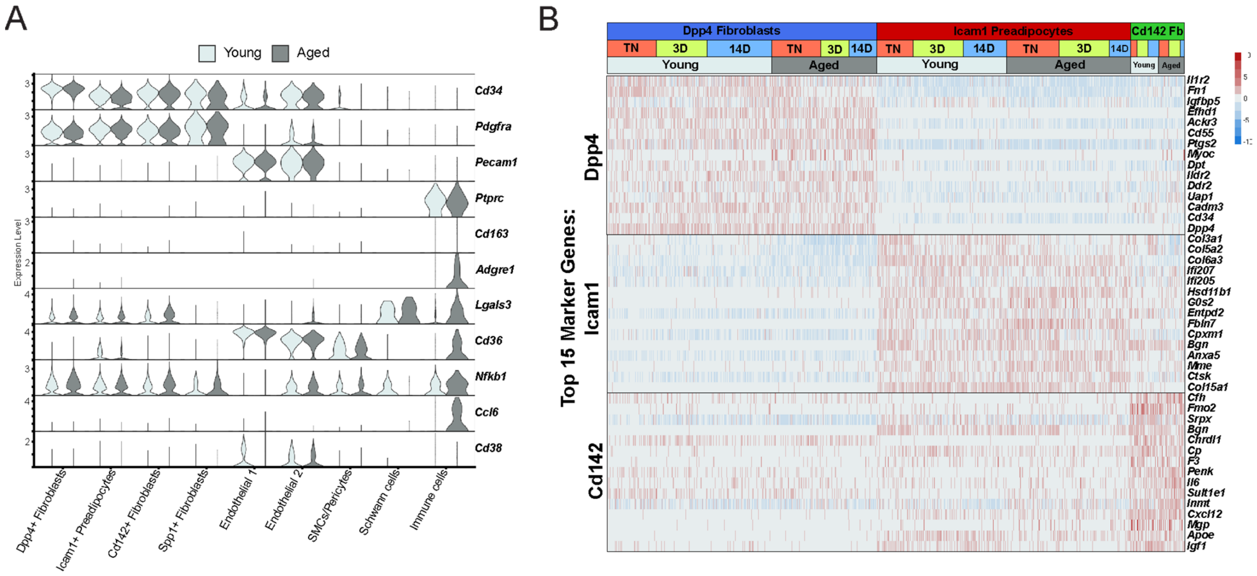


**Figure S3, related to Figure 3.** (**A**) Violin plot showing expression of ARC marker genes in cell clusters split by age, Y-axis = log-scale normalized read count. (**B**) Expression heatmap of top ASPC marker genes across age and housing conditions.

bioRxiv preprint doi: https://doi.org/10.1101/2023.03.20.533514; this version posted February 1, 2024. The copyright holder for this preprint (which was not certified by peer review) is the author/funder, who has granted bioRxiv a license to display the preprint in perpetuity. It is made available under aCC-BY 4.0 International license.

Figure S4

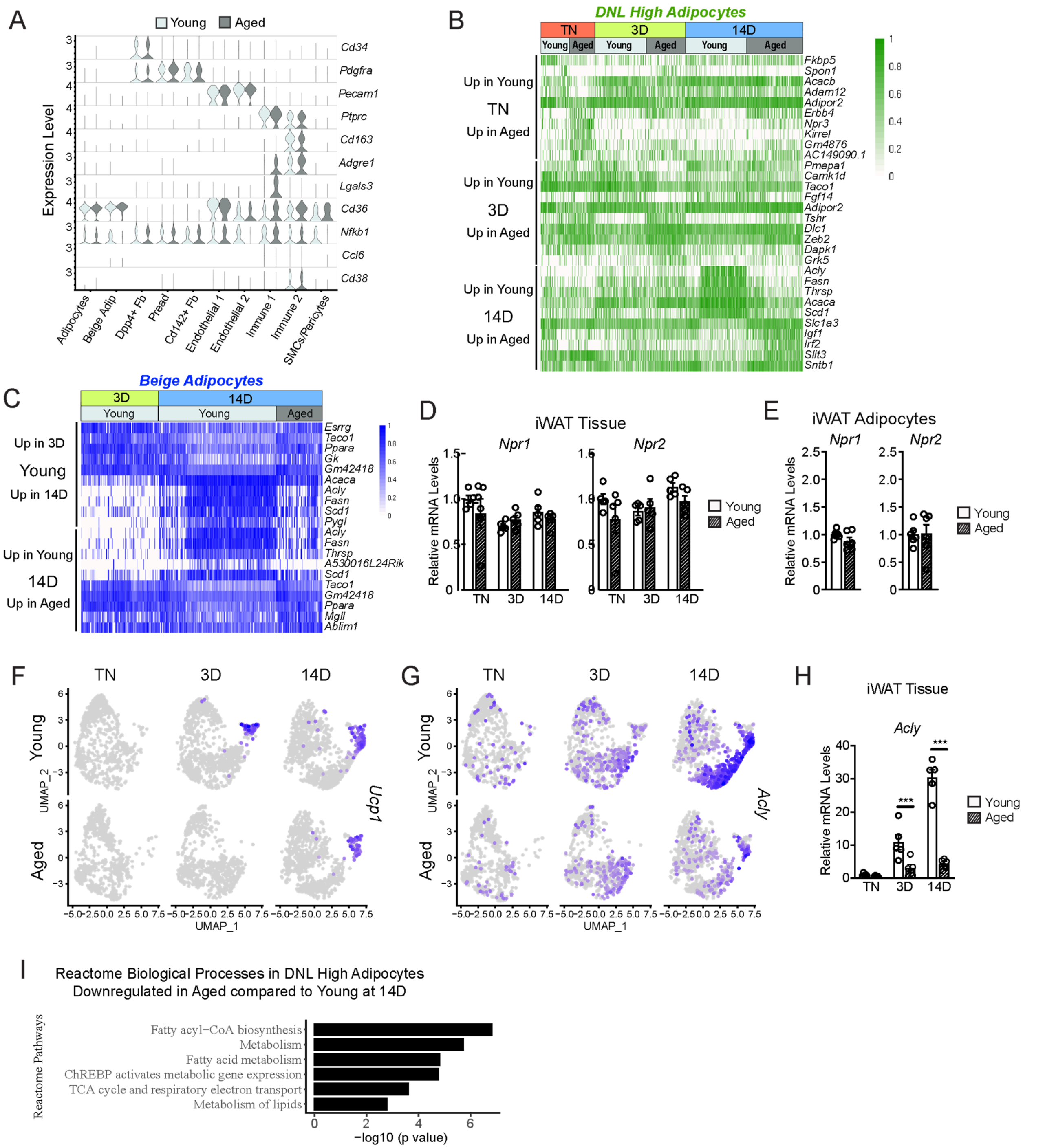


**Figure S4, related to Figures 5,6.** (**A**) Violin plot showing expression levels of ARC marker genes split by age, y-axis = log-scale normalized read count. (**B**) Expression heatmap of the top aging-regulated genes in DNL-high adipocytes. (**C**) Expression heatmap of the top aging-regulated and cold-regulated genes in beige adipocytes. (**D-E**) *Npr1* and *Npr2* mRNA levels in (D) iWAT from mouse groups described in Figure 1A, n=5 and (E) isolated adipocytes from iWAT from TN-acclimated young and aged mice, n=6. (**E-F**) UMAP of *Ucp1* (E) and *Acly* (F) mRNA levels in adipocyte populations (from Figure 5D). (**H**)

bioRxiv preprint doi: https://doi.org/10.1101/2023.03.20.533514; this version posted February 1, 2024. The copyright holder for this preprint (which was not certified by peer review) is the author/funder, who has granted bioRxiv a license to display the preprint in perpetuity. It is made available under aCC-BY 4.0 International license.

*Acly* mRNA levels in iWAT from mouse groups described in Figure 1A, n=5. (**G**) Enrichment analysis displaying the top six Reactome pathways in DNL high adipocytes downregulated in aged at 14 days. Data represent mean ± SEM, points represent biological replicates, 2 groups analyzed using a Student's t-test, and multiple conditions analyzed with a two-way ANOVA with a Tukey correction for multiple comparisons. Significance: not significant, P > 0.05; * P < 0.05 ** P < 0.01; *** P < 0.001.
