# Supplementary material for: Aging impairs cold-induced beige adipogenesis and adipocyte metabolic reprogramming
Source: bioRxiv. 2023 Mar 23:2023.03.20.533514. Preprint. [Version 1] doi: 10.1101/2023.03.20.533514 (PMC10055201; doi:10.1101/2023.03.20.533514)
Supplement: Supplementary file 1 [file NIHPP2023.03.20.533514v1-supplement-1.pdf]

bioRxiv preprint doi: https://doi.org/10.1101/2023.03.20.533514; this version posted March 23, 2023. The copyright holder for this preprint (which was not certified by peer review) is the author/funder, who has granted bioRxiv a license to display the preprint in perpetuity. It is made available under aCC-BY 4.0 International license.

Supplemental Figures

Figure S1

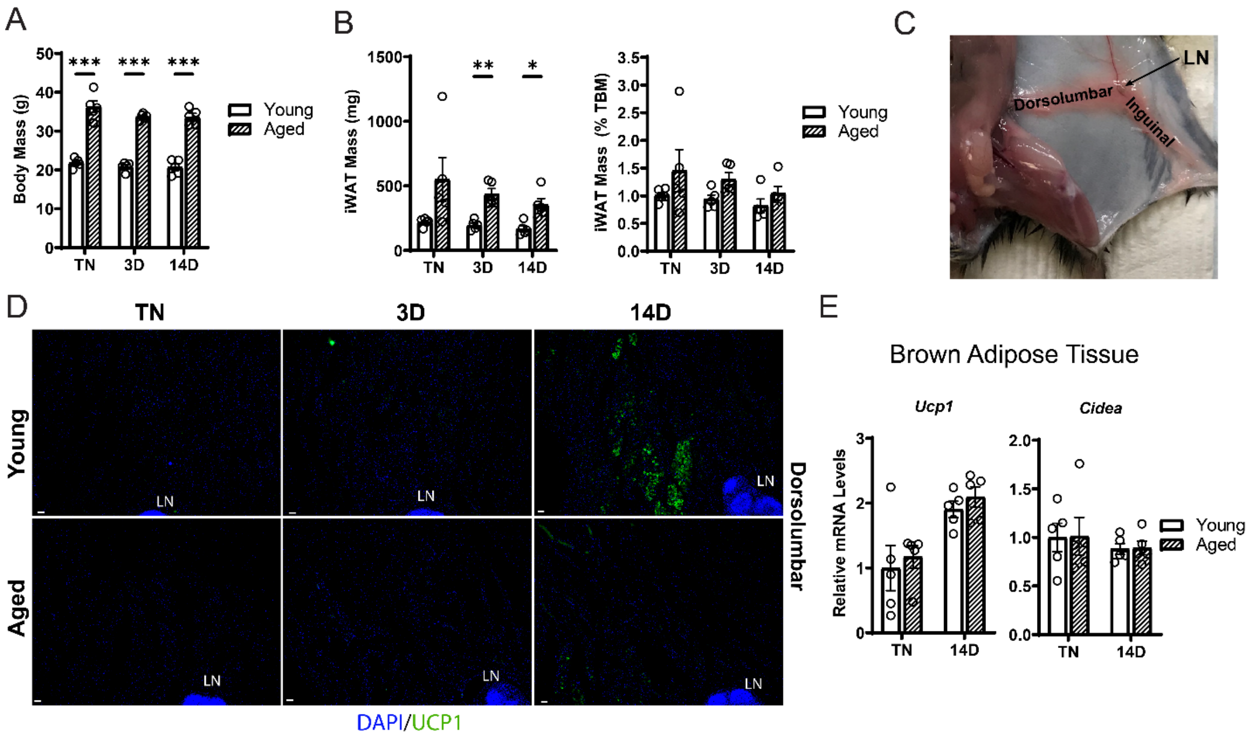

Figure S1, related to Figure 1. (**A-B**) Body mass and iWAT mass of mice described in Figure 1A, n=5. (**C**) Mouse dissection with lymph node (LN) orientation showing the dorsolumbar and inguinal regions of the iWAT pad. (**D**) Immunofluorescence analysis of iWAT with UCP1 (green) and DAPI (blue). LN=lymph node. Scale bar 100 μm. (**E**) mRNA levels of *Ucp1* and *Cidea* in BAT of young and aged mice housed at TN, and either maintained at TN or exposed to cold for 2 weeks. Data represent mean ± SEM, points represent biological replicates, analyzed using a Student's t-test with a Holm-Šidák correction for multiple comparisons. Significance: not significant, P > 0.05; * P < 0.05 ** P < 0.01; *** P < 0.001.

bioRxiv preprint doi: https://doi.org/10.1101/2023.03.20.533514; this version posted March 23, 2023. The copyright holder for this preprint (which was not certified by peer review) is the author/funder, who has granted bioRxiv a license to display the preprint in perpetuity. It is made available under aCC-BY 4.0 International license.

Figure S2

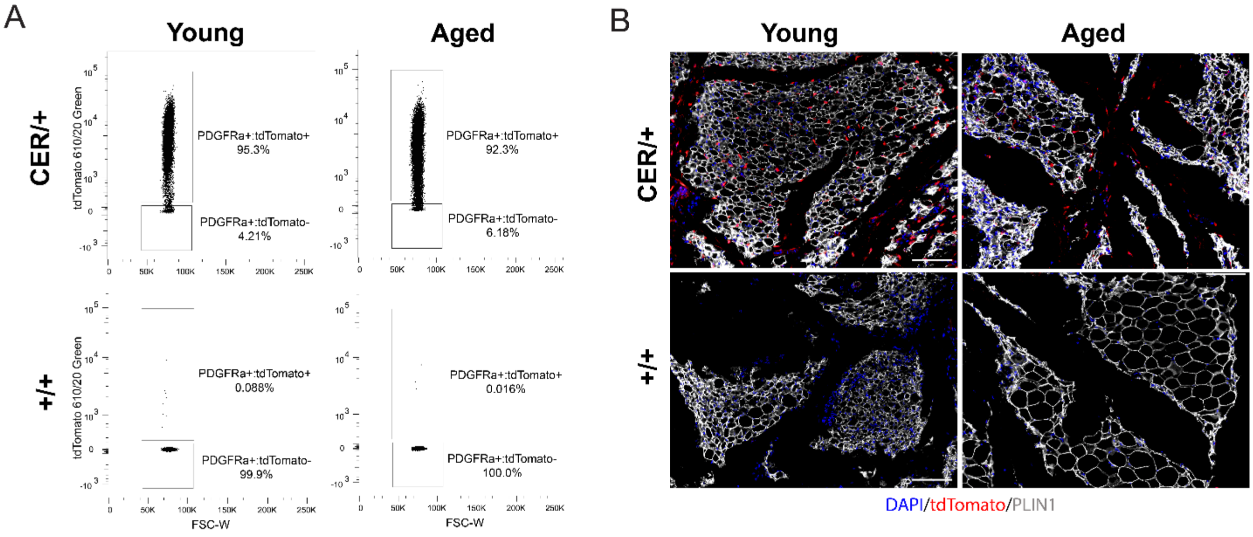

Figure S2, related to Figure 2. (**A**) Representative flow cytometry plots showing expression of tdTomato in gated Live, Lin$^-$; PDGFRα$^+$ stromal vascular cells isolated from young and aged reporter mice (described in Figure 2) immediately after treatment with tamoxifen (tmx, pulse). (**B**) Immunofluorescence analysis of iWAT from young and aged reporter mice with tdTomato (red), PLIN1 (white) and DAPI (blue) after the tmx pulse, scale bar 100 μm.

Figure S3

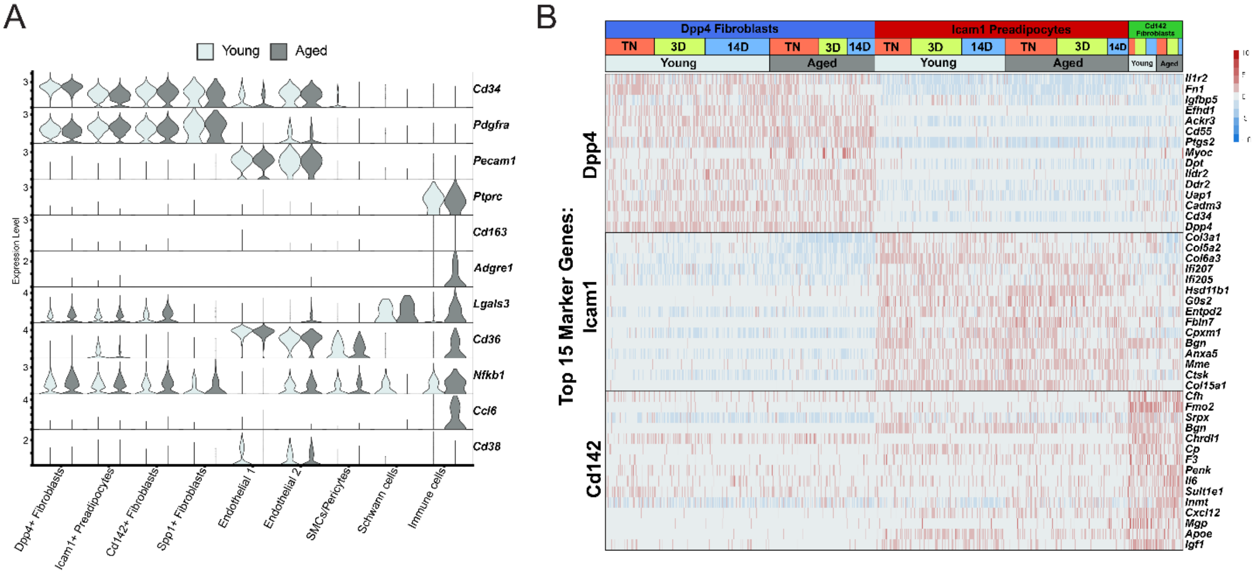

Figure S3, related to Figure 3. (**A**) Violin plot showing expression of ARC marker genes in cell clusters split by age, Y-axis = log-scale normalized read count. (**B**) Expression heatmap of top ASPC marker genes across age and housing conditions.

bioRxiv preprint doi: https://doi.org/10.1101/2023.03.20.533514; this version posted March 23, 2023. The copyright holder for this preprint (which was not certified by peer review) is the author/funder, who has granted bioRxiv a license to display the preprint in perpetuity. It is made available under aCC-BY 4.0 International license.

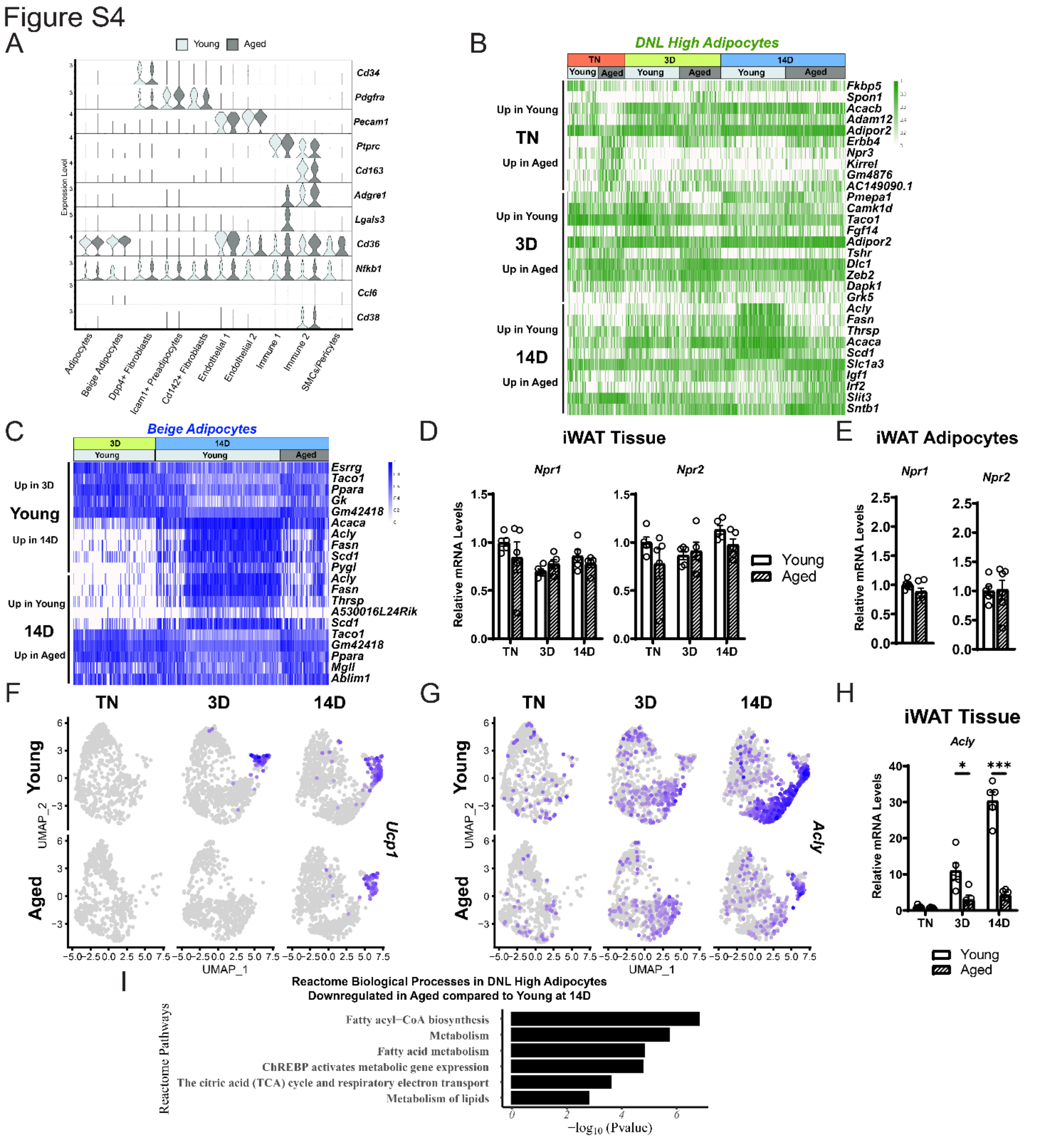

Figure S4, related to Figures 5,6. (**A**) Violin plot showing expression levels of ARC marker genes split by age, y-axis = log-scale normalized read count. (**B**) Expression heatmap of the top aging-regulated genes in DNL-high adipocytes. (**C**) Expression heatmap of the top aging-regulated and cold-regulated genes in beige adipocytes. (**D-E**) *Npr1* and *Npr2* mRNA levels in (D) iWAT from mouse groups described in Figure 1A, n=5 and (E) isolated adipocytes from iWAT from TN-acclimated young and aged mice, n=6. (**E-F**) UMAP of *Ucp1* (E) and *Acly* (F) mRNA levels in adipocyte populations (from Figure 5D). (**H**) *Acly* mRNA levels in iWAT from mouse groups described in Figure 1A, n=5. (**G**) Enrichment analysis displaying the top six Reactome pathways in DNL high adipocytes downregulated in aged at 14 days. Data represent mean ± SEM, points represent

bioRxiv preprint doi: https://doi.org/10.1101/2023.03.20.533514; this version posted March 23, 2023. The copyright holder for this preprint (which was not certified by peer review) is the author/funder, who has granted bioRxiv a license to display the preprint in perpetuity. It is made available under aCC-BY 4.0 International license.

biological replicates, 2 groups analyzed using a Student's t-test, and multiple conditions analyzed with a Holm-Šidák correction for multiple comparisons. Significance: not significant, $P > 0.05$; * $P < 0.05$ ** $P < 0.01$; *** $P < 0.001$.
